# Supplementary material for: Genomic characterization of Pseudomonas syringae pv. syringae from Callery pear and the efficiency of associated phages in disease protection
Source: Microbiol Spectr. 2024 Feb 7;12(3):e02833-23. doi: 10.1128/spectrum.02833-23 (PMC10913373; doi:10.1128/spectrum.02833-23)
Supplement: Supplementary figures — All supplementary figures. [file spectrum.02833-23-s0001.pdf]

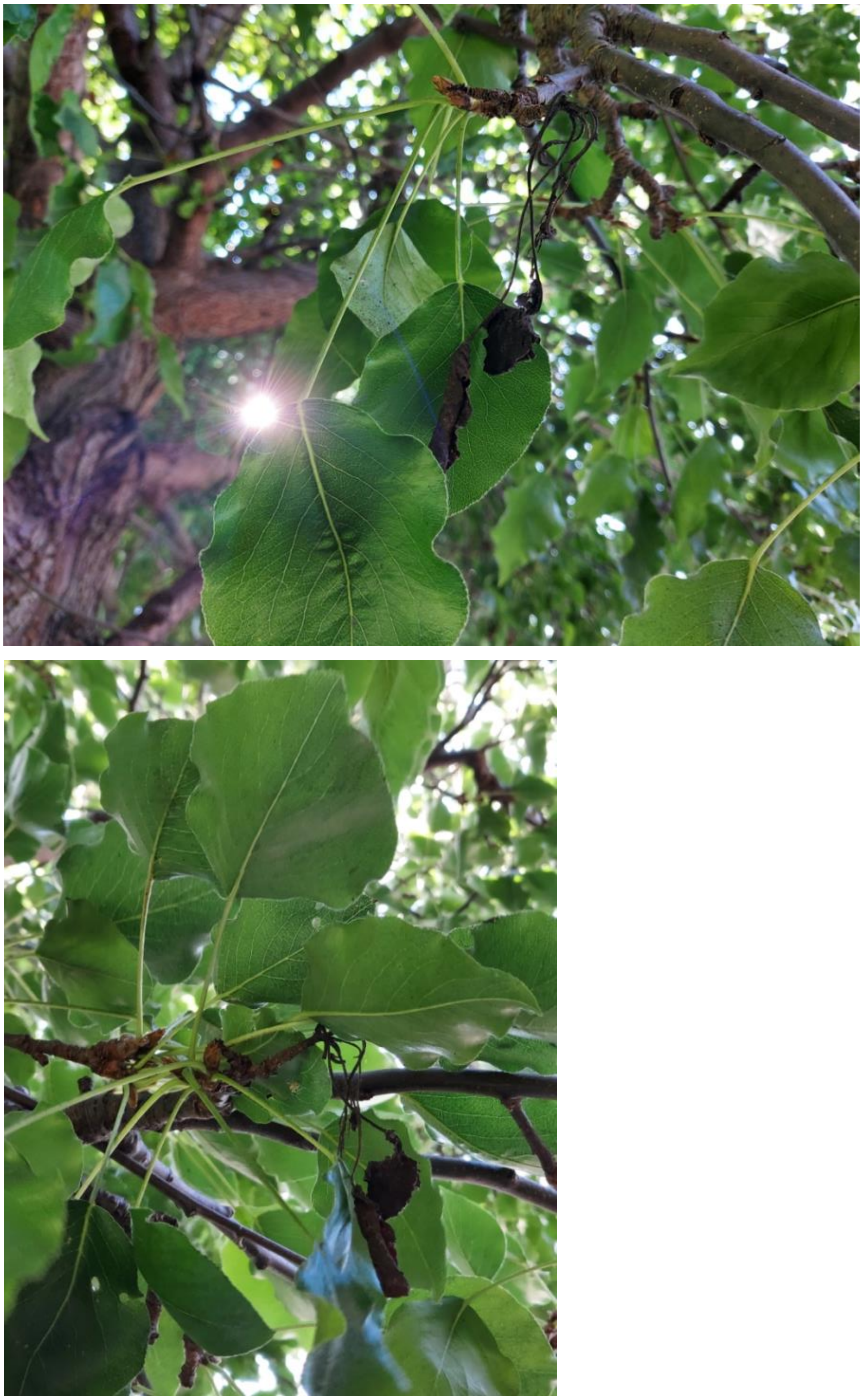

Supplementary Figure 1 Symptoms of blossom blight in Callery pear in Berkeley, California.

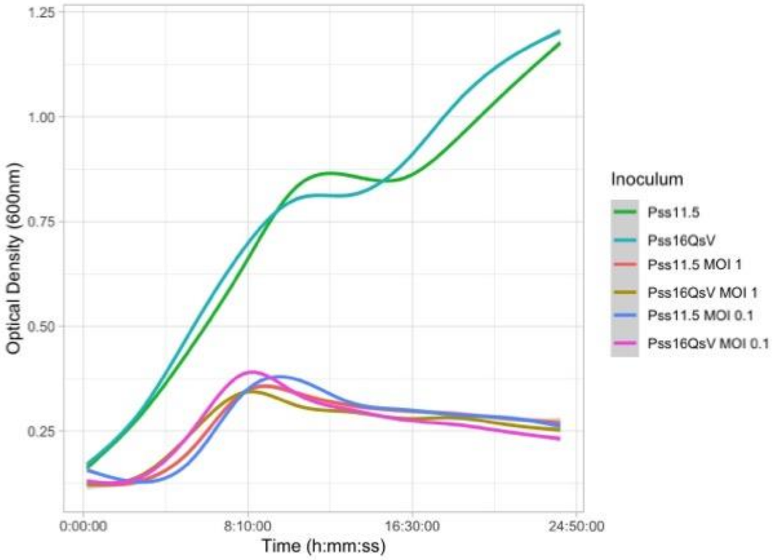

Supplementary Figure 2 Infection curve (OD_{600} in function of time) of phage 16Q on Pss16QsV and Pss11.5. Phages were added at the start of the measurement at different MOI 0.1 and 1. Around eight hours, bacterial concentrations reached a maximum after which the optical density decreased again.

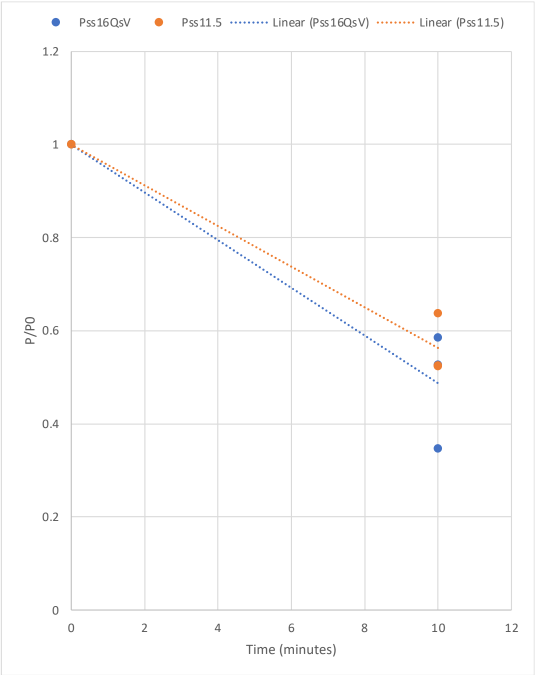

Supplementary Figure 3 Adsorption curve of phage 16Q on Pss16QsV (Blue) and Pss11.5 (orange) expressed as the ratio of the initial phage concentration and the concentration of free phage after ten minutes in function of time. There was no significant difference in the efficiency of adsorption of phage 16Q on the different strains (p-value >0.05).

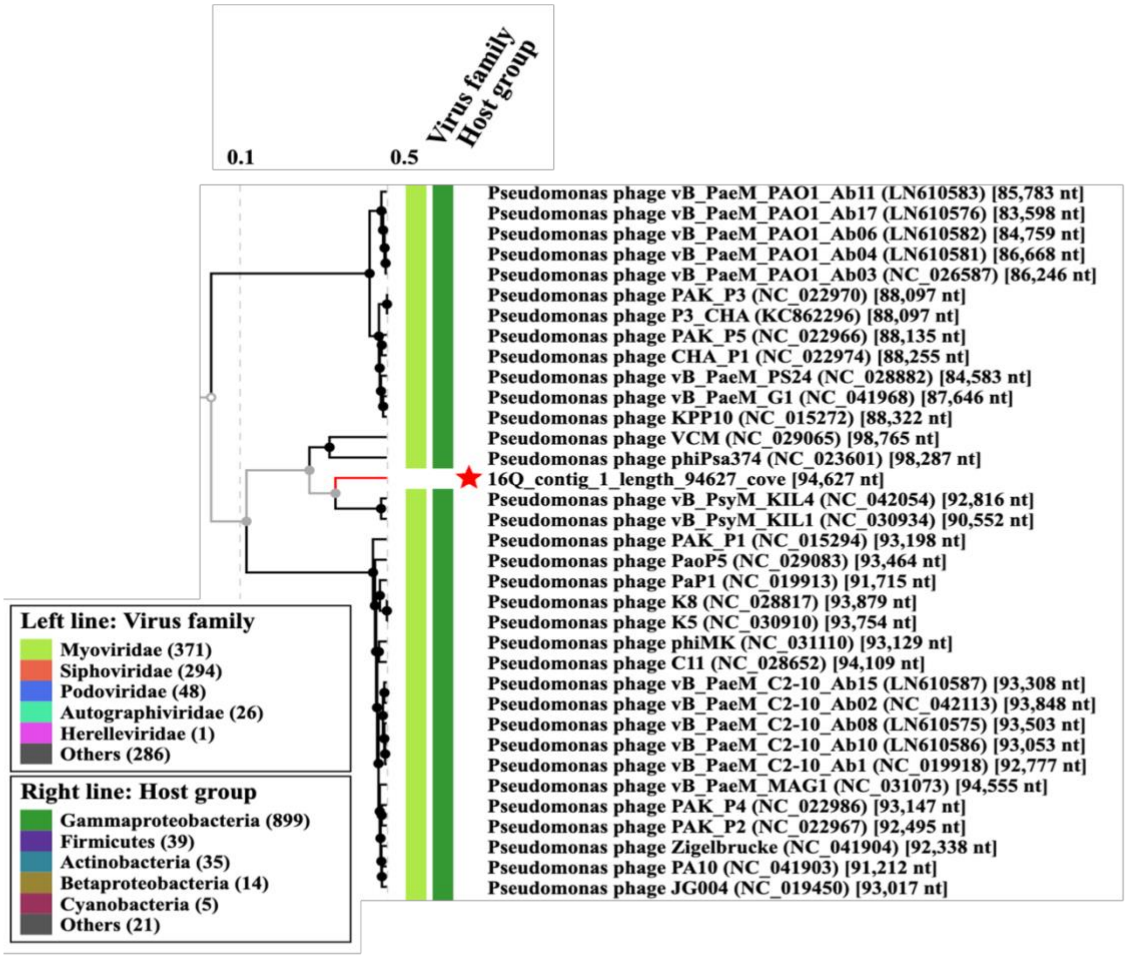

Supplementary Figure 4 VipTree analysis of phage 16Q. the genome clusters together with members of the *Flaumdravirus* genus as well as phage VCM and phiPsa374.

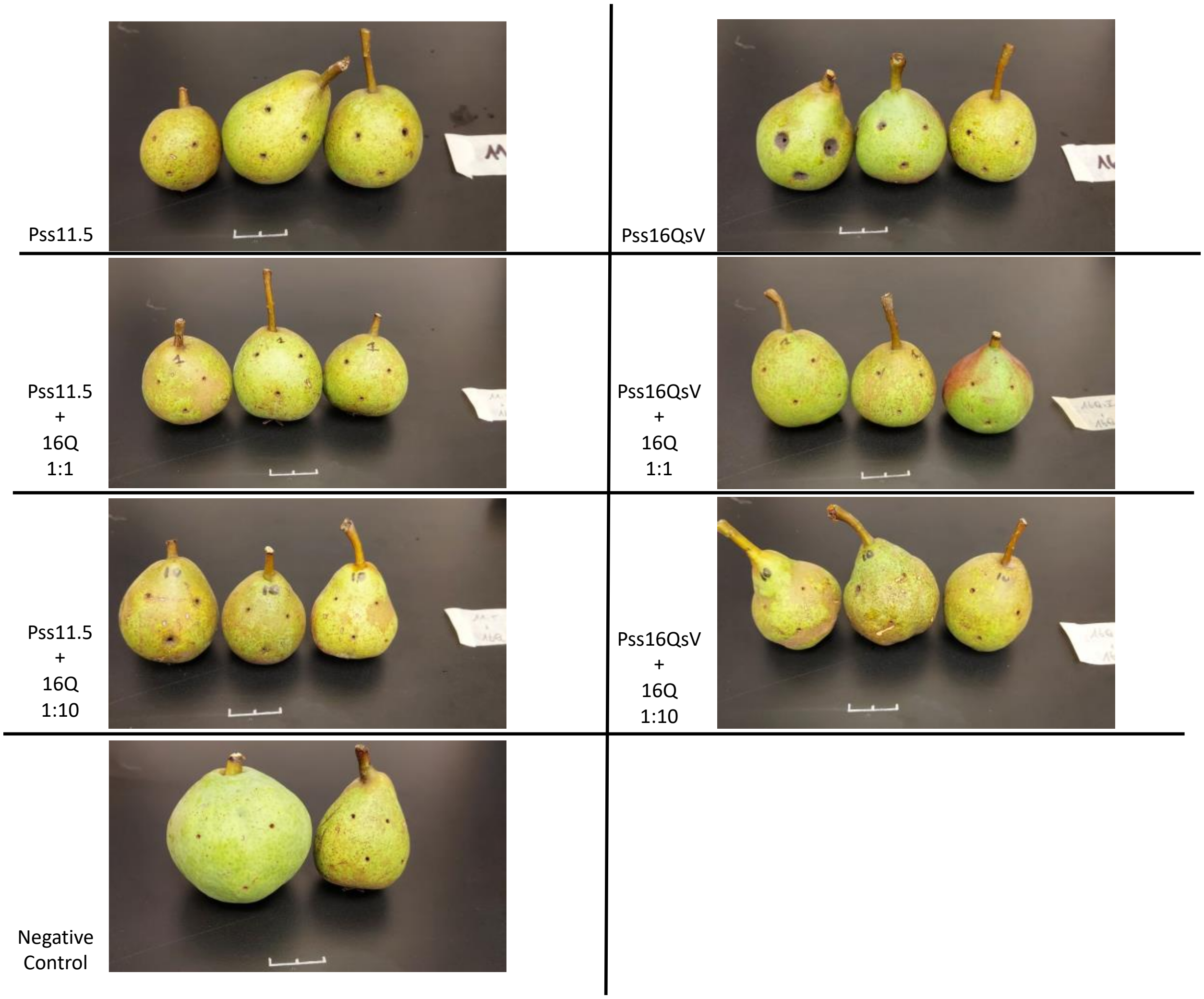

Supplementary Figure 5 Immature pear fruit (commercial pear) with strain Pss11.5 and Pss16QsV with and without phage 16Q in different ratios (1:1 and 1:10). The scale corresponds with 2 cm.

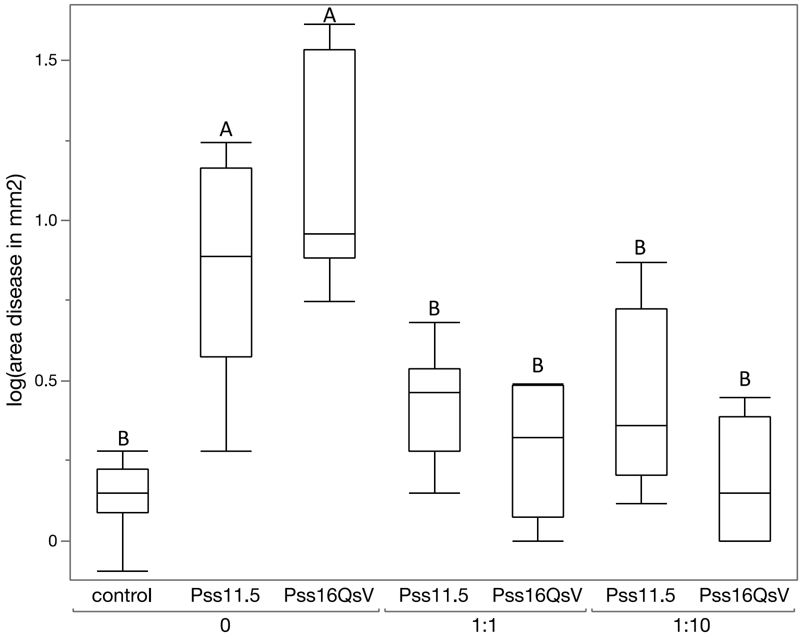

Supplementary Figure 6 Quantile boxplots of the logarithmic area of necrotic tissue (mm^2) around the inoculation sites in immature pear fruits (n = 9). Pss16QsV and Pss11.5 were inoculated with a concentration of 10^8 CFU/mL as well as phage 16Q at a ratio of 1:0 (MOI 0 – no phage), 1:1 (MOI 1) and 1:10 (MOI 10). As negative control, pears were inoculated with PBS. Letters A-B represent different groups of significance (Tukey-Kramer HSD test for multiple comparison, p-value < 0.05).

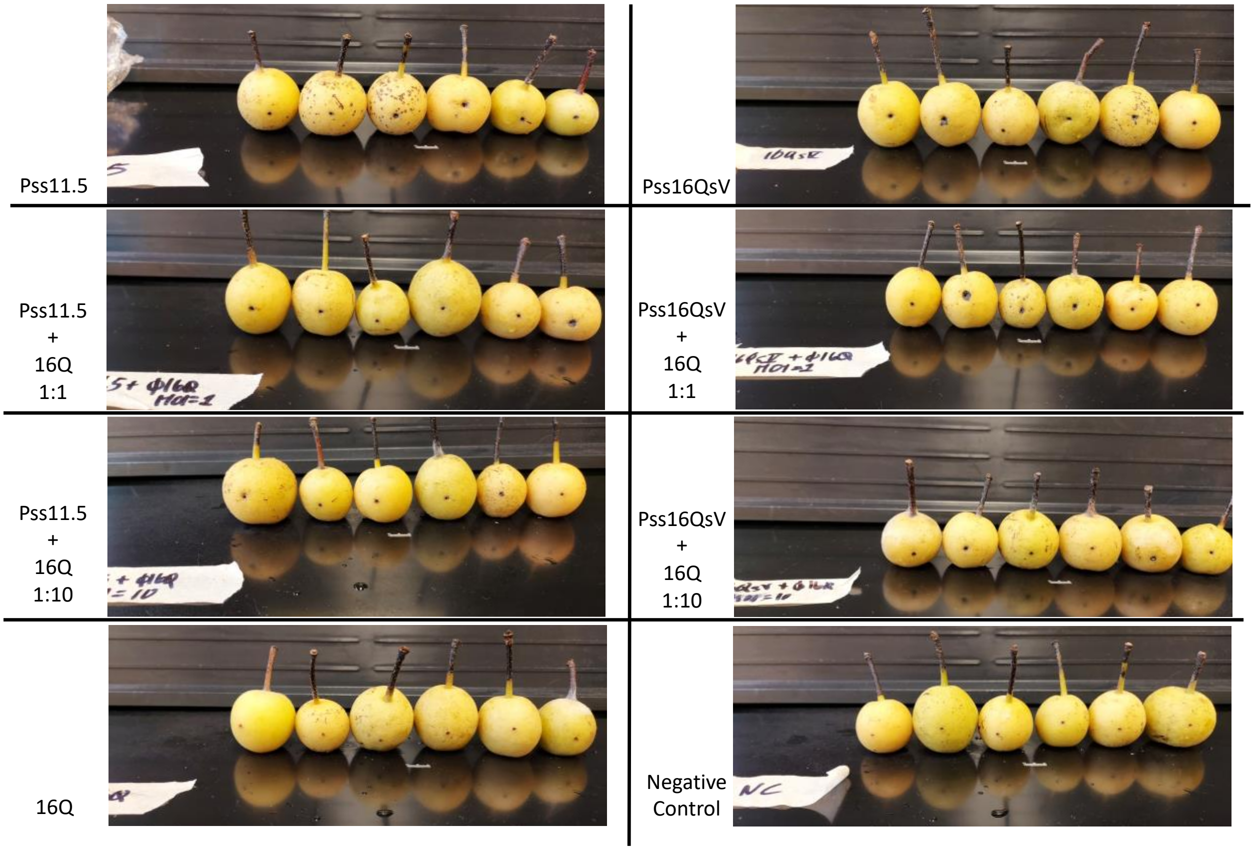

Supplementary Figure 7 Immature pear assay of Asian pear fruit with strain Pss11.5 and Pss16QsV with and without phage 16Q in different ratios (1:1 and 1:10). The scale is 1 cm.
